# Water-enhanced CO_2_ capture in metal–organic frameworks

**DOI:** 10.3389/fchem.2025.1634637

**Published:** 2025-07-08

**Authors:** Celine Cammarere, Jaeden Cortés, T. Grant Glover, Randall Q. Snurr, Joseph T. Hupp, Jian Liu

**Affiliations:** ^1^ School of Chemistry and Materials Science, Rochester Institute of Technology, Rochester, NY, United States; ^2^ School of Medicine and Dentistry, University of Rochester, Rochester, NY, United States; ^3^ Department of Chemical and Biomolecular Engineering, University of South Alabama, Mobile, AL, United States; ^4^ Department of Chemical and Biological Engineering, Northwestern University, Evanston, IL, United States; ^5^ Department of Chemistry and International Institute for Nanotechnology, Northwestern University, Evanston, IL, United States; ^6^ Department of Chemical Engineering, Rochester Institute of Technology, Rochester, NY, United States

**Keywords:** CO_2_ capture, metal-organic framework, nanoporous material, water, humidity

## Abstract

CO_2_ capture from post-combustion flue gas originating from coal or natural gas power plants, or even from the ambient atmosphere, is a promising strategy to reduce the atmospheric CO_2_ concentration and achieve global decarbonization goals. However, the co-existence of water vapor in these sources presents a significant challenge, as water often competes with CO_2_ for adsorption sites, thereby diminishing the performance of adsorbent materials. Selectively capturing CO_2_ in the presence of moisture is a key goal, as there is a growing demand for materials capable of selectively adsorbing CO_2_ under humid conditions. Among these, metal–organic frameworks (MOFs), a class of porous, highly tunable materials, have attracted extensive interest for gas capture, storage, and separation applications. The numerous combinations of secondary building units and organic linkers offer abundant opportunities for designing systems with enhanced CO_2_ selectivity. Interestingly, some recent studies have demonstrated that interactions between water and CO_2_ within the confined pore space of MOFs can enhance CO_2_ uptake, flipping the traditionally detrimental role of moisture into a beneficial one. These findings introduce a new paradigm: water-enhanced CO_2_ capture in MOFs. In this review, we summarize these recent discoveries, highlighting examples of MOFs that exhibit enhanced CO_2_ adsorption under humid conditions compared to dry conditions. We discuss the underlying mechanisms, design strategies, and structural features that enable this behavior. Finally, we offer a brief perspective on future directions for MOF development in the context of water-enhanced CO_2_ capture.

## 1 Introduction

The growing concentration of greenhouse gases, primarily carbon dioxide (CO_2_), in the atmosphere, has led to significant global warming and climate changes. Anthropogenic CO_2_ emissions are largely attributable to the increasing combustion of fossil fuels and various industrial processes designed to satisfy construction, energy, and manufacturing demands. Major contributors include coal- and gas-fired power plants, petrochemical facilities, hydrogen production via steam methane reforming followed by the water-gas shift reaction, and cement manufacturing using calcium carbonate as the primary raw material (Goel et al., 2016; Mukherjee et al., 2019; Dehimi et al., 2025; Park et al., 2021). To keep the atmospheric CO_2_ concentration from rising further, two primary strategies have been pursued. One focuses on developing alternative, clean energy sources that produce little to no CO_2_ (Davis et al., 2018). The other centers on the design of energy-efficient processes for CO_2_ capture, followed by either chemical conversion (Mukherjee et al., 2019; Wang et al., 2017; Sumida et al., 2012; Ran et al., 2018) or geological sequestration (Lin et al., 2024; Massarweh and Abushaikha, 2024; Spurin et al., 2025).

A variety of solid materials have been developed to achieve high CO_2_ uptake and high selectivity for CO_2_ over N_2_, including activated carbons (Jedli et al., 2024), zeolites (Mukherjee et al., 2019; Kumar et al., 2020), metal−organic frameworks (MOFs) (Ghanbari et al., 2020; Schoedel et al., 2016; Lin et al., 2021), polymers (Song et al., 2022), and metal oxides (Yong et al., 2002). Among these, MOFs stand out due to their diverse topologies, large pore volumes, and broadly tunable pore sizes, which can be adjusted by modifying metal nodes and/or organic linkers (Zhou et al., 2012; Horike et al., 2009; Alezi et al., 2016; Wang et al., 2016). Owing to their high surface area, large pore volume and particularly the presence of a high density of open metal sites (Britt et al., 2009), certain MOFs have demonstrated excellent CO_2_ uptake at room temperature. However, high CO_2_ capacity and selectivity in the presence of N_2_ are not enough, as CO_2_ typically coexists with other components, water vapor being one of the most challenging (Siegelman et al., 2019). Water and CO_2_ often target the same adsorption sites, with water typically binding more strongly, thus outcompeting CO_2_ and reducing uptake capacity. This competition becomes critical in applied CO_2_ capture scenarios. For instance, flue gas from natural gas combined cycle (NGCC) power plants contains approximately 75% N_2_, 4% CO_2_, 12% O_2_, and 9% H_2_O by volume (Siegelman et al., 2019; Zhang et al., 2020). At this concentration, water vapor can significantly impair CO_2_ capture by blocking adsorption sites, and must, therefore, be carefully considered in material design and application.

The earliest systems developed for selective CO_2_ capture in the presence of water were aqueous amine solutions, which rely on acid-base reactions to form carbamates (Li and Keeners, 2016). However, these liquid-phase systems suffer from several limitations, including low working capacities, high regeneration energies, and thermal instability (Zhao et al., 2012). To address these issues, researchers developed molecularly porous solid systems incorporating amine functionalities to enhance the selective adsorption of CO_2_ in the presence of water. These approaches include grafting amine groups onto porous materials, such as porous polymers, silica, alumina, and carbon (Filburn et al., 2005; Wurzbacher et al., 2011; Kuwahara et al., 2012; Chai et al., 2016; Li, et al., 2010), as well as functionalizing MOFs with diamine-containing molecules (Choe et al., 2019; Li et al., 2015; Kang et al., 2019; Choi et al., 2012; Demessence et al., 2009; McDonald et al., 2011; Liao et al., 2016; McDonald et al., 2012; Planas et al., 2013; McDonald et al., 2015; Siegelman et al., 2017; Milner et al., 2018; 2017). In diamine-appended MOFs, one end of the diamine molecule binds to an open metal site in the MOF, while the other end remains available for CO_2_ chemisorption. These diamine-functionalized MOFs introduce chemisorption sites, enhancing selectivity for CO_2_ over H_2_O. However, their overall CO_2_ uptake capacity and uptake kinetics may be affected, as the diamine molecules partially occupy the available pore volume.

Several studies have now demonstrated that interactions between water and CO_2_ within confined pore spaces can, in fact, enhance CO_2_ capture. These findings represent a new paradigm, revealing that certain MOFs can convert the traditionally negative impact of moisture into a beneficial factor for improving CO_2_ adsorption performance. This review highlights these key discoveries and examines the unique mechanisms underlying enhanced CO_2_ adsorption under humid conditions. We conclude with a brief perspective on future directions for MOF design and research in the field of water-enhanced CO_2_ capture.

## 2 Water-enhanced CO_2_ capture

### 2.1 Dipole–quadrupole interaction

In a high-throughput screening study (Chanut et al., 2017), Chanut et al. investigated the effect of pre-equilibrated water on CO_2_ uptake in 45 MOFs using thermogravimetric analysis. The MOFs were grouped into various categories based on the extent to which pre-adsorbed H_2_O influenced CO_2_ uptake. One category, which included MIL-110(Al), MIL-163(Zr), Cu-HKUST-1 and UiO-66(Zr) (Table 1), exhibited a slight increase in CO_2_ uptake with a certain amount of pre-adsorbed water. For example, Cu-HKUST-1 showed an approximately 5 wt% increase in CO_2_ uptake in the presence of 2–4% relative humidity (RH). This observation is consistent with Yazaydin’s report that CO_2_ uptake and its selectivity over N_2_ increased in 4 wt% hydrated Cu-HKUST-1 due to the presence of water molecules coordinated to the framework open-metal sites (Yazaydın et al., 2009). This enhancement was initially predicted through molecular simulations and later validated by experiments. Detailed examination of interaction energies using grand canonical Monte Carlo simulations suggested that Coulombic interactions are responsible for the increased CO_2_ adsorption–specifically interactions between the quadrupole moment of CO_2_ and the electric field generated by water molecules bound to open metal sites. The LeVan group reported similar findings for Cu-HKUST-1 through volumetric measurements (Liu et al., 2010). Collectively, these results suggest an unexpected approach for enhancing CO_2_ capture in the presence of water. However, at high humidity levels, Cu-HKUST-1 undergoes structural degradation, which likely explains why enhanced CO_2_ uptake was not observed under conditions of high RH. Yu et al. (2016) investigated the effect of water on CO_2_ capture in an isostructural series of M-HKUST-1 frameworks (M = Zn, Co, Ni, and Mg) through simulation studies evaluating water coordination within the MOFs. Water-coordination enhanced CO_2_ uptake, similar to that observed in Cu-HKUST-1, was found for the Zn-, Co-, and Ni-based analogues. However, for Mg-HKUST-1, water coordination reduced CO_2_ adsorption at higher pressures.

**TABLE 1:** MOFkey and MOFid codes for some MOFs mentioned in this paper (Bucior et al., 2019).

MOF Name	MOFkey	MOFid
Cu-HKUST-1	Cu.QMKYBPDZANOJGF.MOFkey-v1.tbo	[Cu][Cu].[O-]C(═O)c1cc(cc(c1)C(═O)[O-])C(═O)[O-] MOFid-v1.tbo.cat0
UiO-66	Zr.KKEYFWRCBNTPAC.MOFkey-v1.fcu	[O-]C(═O)c1ccc(cc1)C(═O)[O-].[O]12[Zr]34[OH]5[Zr]62[OH]2[Zr]71[OH]4[Zr]14[O]3[Zr]35[O]6[Zr]2([O]71)[OH]43 MOFid-v1.fcu.cat0
MIL-100	Cr.QMKYBPDZANOJGF.MOFkey-v1.moo	F[Cr][O]([Cr])[Cr].F[Cr][O]([Cr]F)[Cr].[Cr][O]([Cr])[Cr].[O-]C(═O)c1cc(cc(c1)C(═O)[O-])C(═O)[O-] MOFid-v1.moo.cat0
Mg-MOF-74	Mg.YXUXCIBWQAOXRL.MOFkey-v1.UNKNOWN	[Mg].[O-]C(═O)c1cc([O])c(cc1[O])C(═O)[O-] MOFid-v1.UNKNOWN.cat0
MOF-808	Zr.QMKYBPDZANOJGF.MOFkey-v1.spn	O[Zr]123([OH2])[OH]4[Zr]56([O]3[Zr]37([OH]2[Zr]28([O]1[Zr]14([O]6[Zr]([OH]53)([OH]21)([O]78)([OH2])O)([OH2])(O)O)[OH2])([OH2])(O)O)[OH2].[O–]C(O)c1cc(cc(c1)C(O)[O–])C(O)[O–] MOFid-v1.spn.cat0
NOTT-400	Sc.QURGMSIQFRADOZ.MOFkey-v1.UNKNOWN	[O-]C(=O)c1cc(cc(c1)C(=O)[O-])c1cc(cc(c1)C(=O)[O-])C(=O)[O-].[OH].[Sc] MOFid-v1.UNKNOWN.cat0
Mg-CUK-1	Mg.WAYLQVWVRREMCZ.MOFkey-v1.UNKNOWN	[Mg].[O-]C(=O)c1ccc(cc1)c1ccnc(c1)C(=O)[O-].[OH] MOFid-v1.UNKNOWN.cat0

**FIGURE 1:**
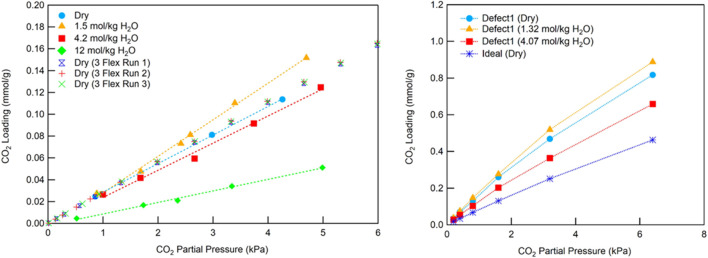
Binary CO_2_/H_2_O adsorption isotherms for UiO-66 at various fixed water loadings, compared to the dry condition. (Left) Experimental results; (Right) Simulation studies. Reprinted with permission from Hossain et al. (2019). Copyright 2019, Elsevier Ltd.

Another water-stable MOF, UiO-66, was reported by Hossain et al. to show a similar result, exhibiting a slight enhancement in CO_2_ adsorption at low water loading (1.5 mol/kg) under low CO_2_ partial pressure (below 5 kPa) at 25°C. This observation was based on experimental binary adsorption isotherms measured volumetrically using a mass balance approach (Hossain et al., 2019). However, increasing the co-adsorbed water loadings to 4.2 and 12 mol/kg led to reduced CO_2_ uptake (Figure 1 Left). Molecular simulations supported these findings and further revealed that the effect depends on the type of defect sites within the MOF: missing linker defects promoted the enhancement (Figure 1 Right), whereas missing cluster defects didn’t show this behavior. Expanding on this work, Hernandez et al. conducted computational studies on three amine-linker UiO-66 materials and found that water molecules bridge between metal-oxide clusters by occupying missing linker positions (Hernandez et al., 2021). These water bridges reduce the pore size in defect-laden MOFs and enhance CO_2_ adsorption in the presence of co-adsorbed water. Experimental binary isotherm data were consistent with these predictions. These studies underscore the importance of considering defect sites when evaluating CO_2_ capture performance in humid conditions.

### 2.2 H_2_O dissociation leading to new adsorption sites

**FIGURE 2:**
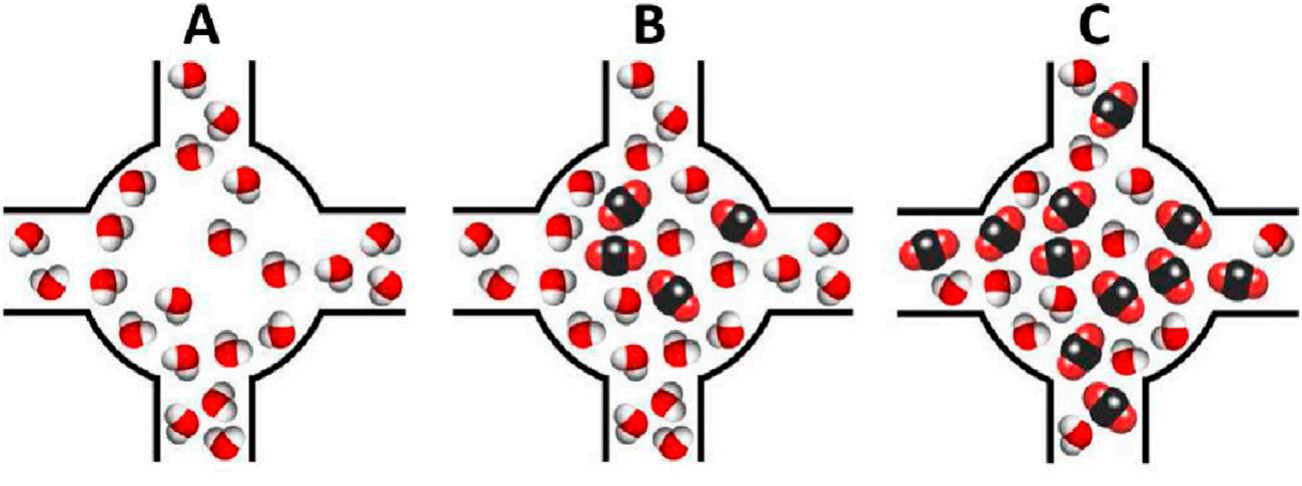
Schematic representation of the water channel formed in MIL-100(Fe) to enhance the CO_2_ adsorption **(A)** Adsorbed water molecules create pockets that can **(B)** adsorb CO_2_ molecules, which can in turn **(C)** displace some of the water molecules. Reprinted with permission from Soubeyrand-Lenoir et al. (2012). Copyright 2012, American chemical society.

MIL-100(Fe) was evaluated by Soubeyrand-Lenoir et al., who reported a five-fold increase in CO_2_ uptake (105 mg/g) at low pressure (200 mbar) under moderate humidity (40% RH) (Soubeyrand-Lenoir et al., 2012). They hypothesized that water molecules coordinate to the Lewis-acidic metal sites, forming water channels, while CO_2_ adsorption occurs in the center of these channels without carbonate formation. In addition to the water stability of the materials, its mesoporosity was highlighted as a key factor contributing to the observed enhancement, as it allows for the formation of microporous water pockets that can subsequently be filled with CO_2_ (see Figure 2).

**FIGURE 3:**
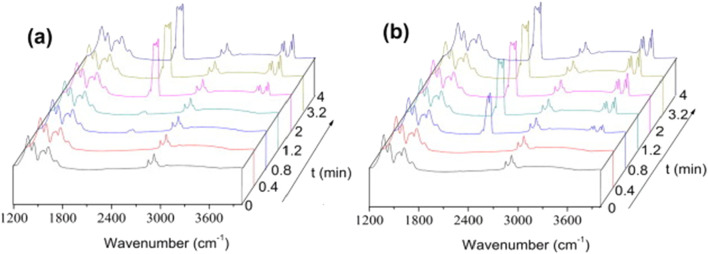
*In situ* IR spectra of MIL-100(Fe) loaded with CO_2_ in the spectral region between 1,200 and 4,000 cm^−1^. **(a)** CO_2_ gas flow rate 40 mL/min, 298 K in dry condition. **(b)** CO_2_/H_2_O gas flow rate 40 mL/min, 298 K, 50% RH. Reprinted with permission from Xian et al. (2015). Copyright 2015, Elsevier Ltd.

Xian et al. further investigated this idea in MIL-100(Fe) using CO_2_ temperature programmed desorption (TPD) and *in situ* Fourier transform infrared spectroscopy (FTIR) (Xian et al., 2015). TPD measurements revealed two CO_2_ desorption peaks in the hydrated sample (50% RH), compared to only one in the dehydrated sample, indicating the creation of an additional adsorption site in the presence of water. The authors proposed that water molecules dissociate to form node hydroxyl groups, which serve as extra adsorption sites for CO_2_, thereby leading to an additional adsorption site to enhance the material’s uptake capacity. *In situ* FTIR results supported this conclusion by revealing faster CO_2_ adsorption under humid conditions, as evidenced by the more rapid appearance of CO_2_ stretching mode peaks (see peaks at 2,350, 3,600, and 3,700 cm^−1^ in Figure 3). The water dissociation hypothesis is worthy of further investigation.

### 2.3 Water nanopocket confinement effects

For MOFs containing bridging hydroxo ligands as components of nodes, computational studies have predicted that at low water loadings, H_2_O molecules can be efficiently packed through strong hydrogen bonding to the–OH groups (Liu et al., 2023; He et al., 2023). These well-ordered water molecules can, in turn, improve CO_2_ adsorption by forming favorable hydrogen bonds with CO_2_ within the microchannels. In other words, the hydroxo ligands act as directing agents for efficient water arrangement, and the pre-adsorbed water molecules introduce confinement effects that further promote CO_2_ uptake.

Building on this principle, the Ibarra group investigated a series of MOFs containing µ_2_/µ_3_-OH ligands, including NOTT-400 (Gonzalez et al., 2015), NOTT-401 (Lara-García et al., 2015; Sánchez-González et al., 2016), InOF-1 (Peralta et al., 2015), and Mg-CUK-1 (Sagastuy-Breña et al., 2018), and demonstrated that their CO_2_ capture capacities were enhanced to varying degrees under moderate humidity (RH < 40%) at 30°C. In a combined experimental and computational study, Breña et al. further reported humidity-enhanced CO_2_ adsorption in Mg-CUK-1, a framework featuring one-dimensional microporous channels (Sagastuy-Breña et al., 2018). Using static CO_2_ adsorption isotherms and thermogravimetric analysis under a constant CO_2_ flow (60 mL/min), they observed a maximum CO_2_ uptake of 8.5 wt% at 18% RH, compared to 4.6 wt% under dry conditions. However, beyond 20% RH, a rapid decline in CO_2_ adsorption was observed, with almost negligible CO_2_ uptake at 25% RH.

**FIGURE 4:**
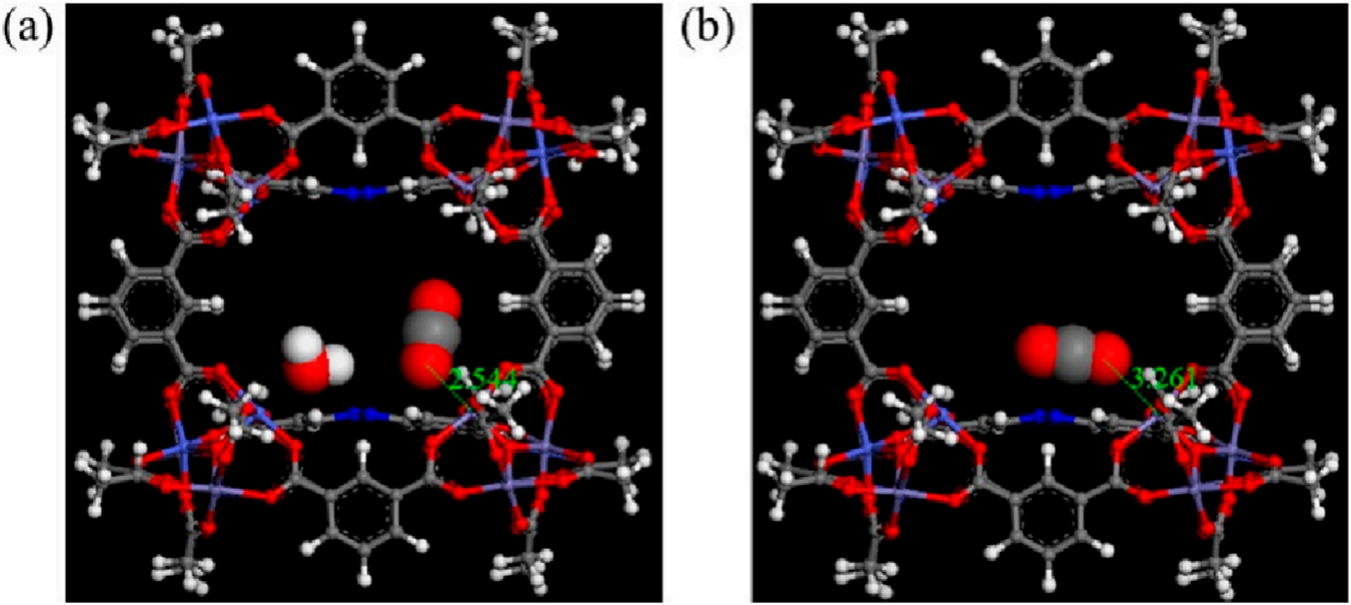
Binding sites of CO_2_ in PCN-250(Fe_2_Co) structure **(a)** with and **(b)** without H_2_O. Reprinted with permission from Chen et al. (2018). Copyright 2018, American chemical society.

Chen et al. (2018) reported unusual moisture-enhanced CO_2_ adsorption in PCN-250(Fe_3_) and PCN-250(Fe_2_Co). These compounds are constructed from trimetallic-oxy clusters, i.e., Fe_3_(μ_3_-O)(CH_3_COO)_6_ or Fe_2_Co(μ_3_-O)(CH_3_COO)_6_, as nodes, and ABTC^4-^ units as linkers (H_4_ABTC = 3,3′,5,5′-azobenzenetetracarboxylic acid). For PCN-250(Fe_3_), the uptake of CO_2_ increases by 54% under 50% RH, compared to dry conditions (from 1.18 to 1.82 mmol/g). PCN-250(Fe_2_Co) exhibited a 69% increase in CO_2_ uptake under the same conditions (from 1.32 to 2.23 mmol/g). Even at 90% RH, significant increases in CO_2_ adsorption were observed (44% for PCN-250(Fe_3_) and 70% for PCN-250(Fe_2_Co)) compared to their respective uptakes under dry conditions. Molecular simulations revealed that node-based, bridging oxo ions (μ_3_-O) act as directing agents for H_2_O adsorption. These water molecules, in turn, help position CO_2_ molecules closer to metal centers on the opposite side of the pore, enhancing CO_2_ adsorption via confinement effects. The CO_2_/MOF interaction is further strengthened by what the authors term a “plier effect”, where coordinated water molecules appear to “clamp” CO_2_ molecules onto open metal sites (see Figure 4), increasing the efficiency of adsorption under unsaturated conditions by maximizing the use of available adsorption sites. The plier effect, involving CO_2_, H_2_O, and the MOFs, enables effectively use of more of the candidate adsorption sites by CO_2_; in turn, the amount of CO_2_ adsorbed increases. The mechanism behind the enhanced CO_2_ uptake at 90% RH is less clear, as such high humidity would presumably saturate the pores with water, leaving little space for CO_2_.

**FIGURE 5:**
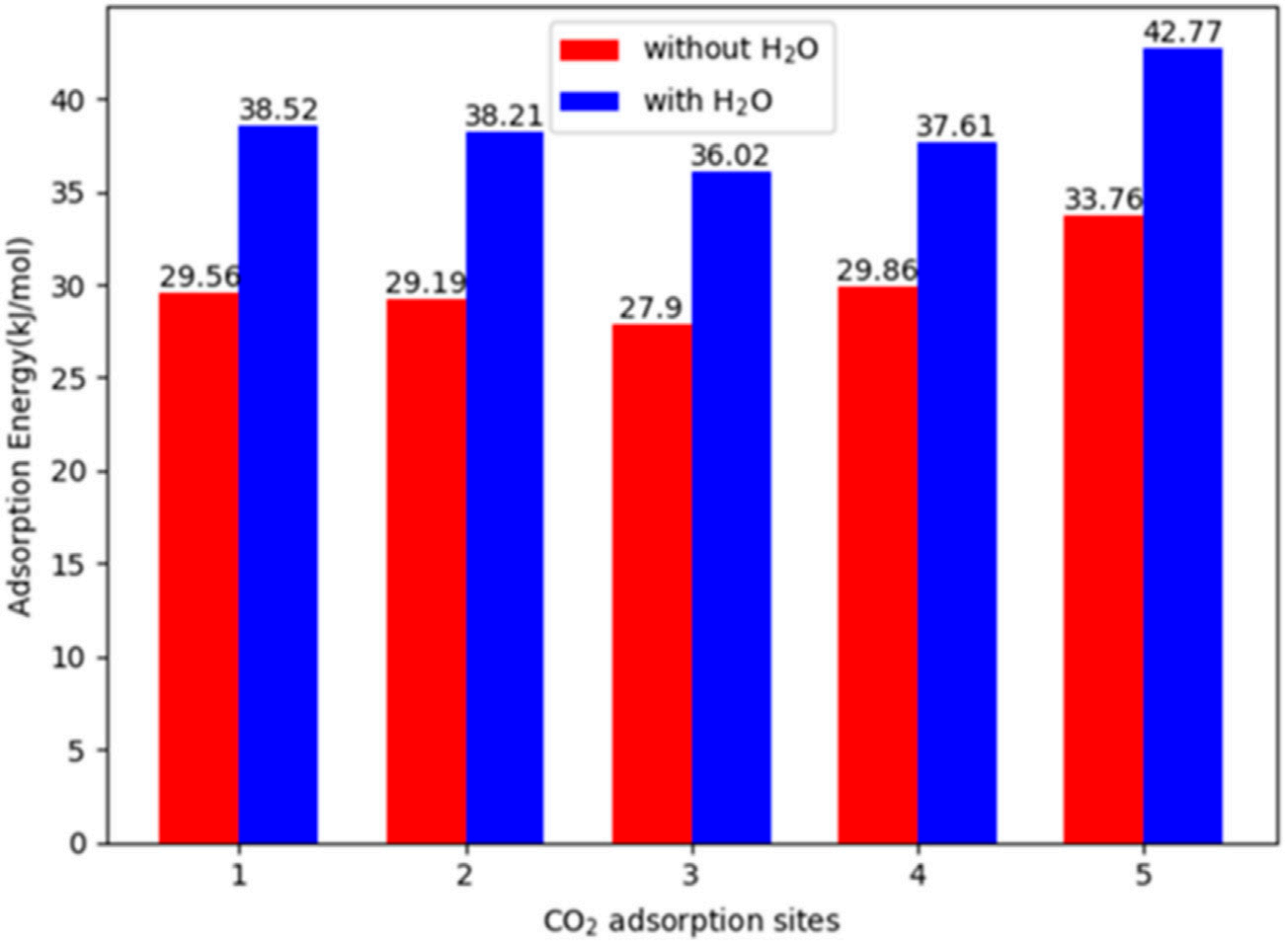
Comparison between CO_2_ adsorption energies without H_2_O and with H_2_O for five distinct CO_2_ adsorption sites in ZnF(daTZ). Reprinted with permission from Shi et al. (2020). Copyright 2020, American chemical society.

Shi et al. (2020) reported a series of metal-triazolate MOFs, constructed from ZnF rods and 1,2,4 triazolate linkers functionalized with various groups (*e.g.*, -NH_2_ and -CH_3_), among which the MOF featuring a 3,5-diamino-1,2,4-triazolate linker (ZnF(daTZ)) exhibited a CO_2_/N_2_ thermodynamic adsorption selectivity of 120 at 298K and 0–101 kPa, and a CO_2_/H_2_O kinetic adsorption selectivity of 70 at 298K and 33% RH. DFT calculations revealed a 25%–30% increment in the heat of CO_2_ adsorption in the presence of co-adsorbed water, indicating stronger CO_2_ binding under humid conditions (see Figure 5). This enhancement was attributed to the preferential localization of water and CO_2_ molecules within the MOF framework, *i.e.*, water molecules tended to occupy the corner sites, while CO_2_ molecules were primarily located at the center of the channels. However, this study does not fully elaborate on how this spatial distribution contributes to the enhanced CO_2_ adsorption under humid conditions. A similar spatial preference was reported in amine-functionalized UiO-66, where H_2_O and CO_2_ adsorbed at different sites (Hernandez et al., 2021). The authors proposed that water molecules formed hydrogen-bonded bridges between metal nodes by occupying missing linker positions, effectively reducing the pore size and enhancing CO_2_ adsorption.

### 2.4 Ammonium carbamate, carbamic acid and bicarbonate formation

Functionalizing MOFs with diamines allows one amine group to coordinate to an open metal site, while the other points into the pore to interact with CO_2_ molecules. For instance, diamine-modified MOF-74 materials have displayed selective CO_2_ adsorption over water due to the formation of carbamate species, particularly at low CO_2_ concentrations (Milner et al., 2018). However, carbamate formation typically requires two amine groups to react with one single CO_2_ molecule, which limits the overall CO_2_ uptake capacity.

**FIGURE 6:**
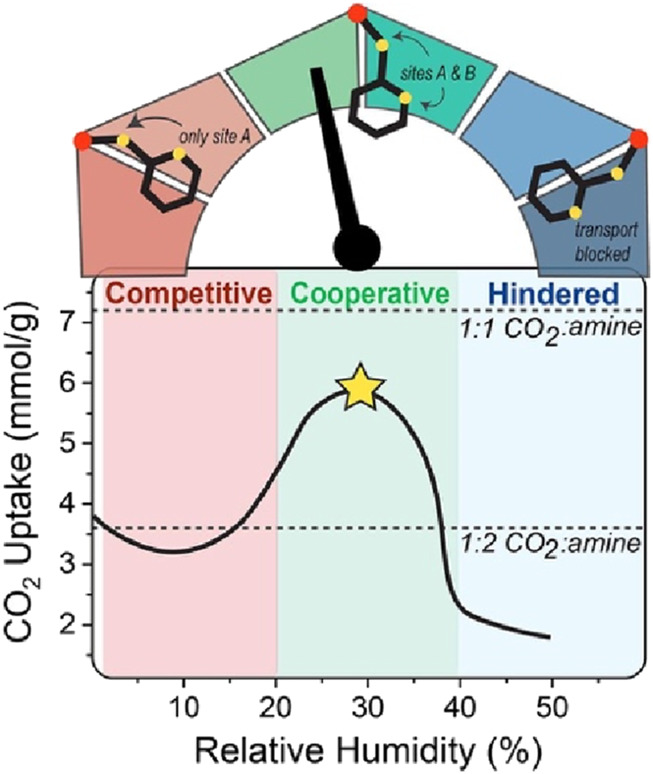
Three distinct RH regions for CO_2_ uptake by (2-ampd)_2_Mg_2_(dobpdc): competitive adsorption from 0%–20% RH, enhanced adsorption between 20%–40% RH, and hindered adsorption at RH levels above 40% at 1 bar and 40°C. Reprinted with permission from Holmes et al. (2023). Copyright 2023, Elsevier Ltd.

The impact of RH on the performance of amine-appended MOFs remains relatively underexplored, with few studies reporting CO_2_ uptake across a broad range humidity levels. Holmes et al. (2023) investigated this effect on (2-ampd)_2_Mg_2_(dobpdc) MOF (2-ampd is 2-(aminomethyl)piperidine) using both gravimetric and breakthrough adsorption techniques. Their findings identified three distinct RH regions based on the influence of water on CO_2_ uptake: competitive adsorption from 0%–20% RH, enhanced adsorption between 20%–40% RH, and hindered adsorption due to pore saturation at RH levels above 40%, see Figure 6. A significant enhancement in CO_2_ uptake at 1 bar and 40°C was observed at 30% RH (5.7 ± 0.2 mmol/g), compared to 3.35 mmol/g under dry conditions, see Figure 6. This increase was attributed to a mixed adsorption mechanism, wherein CO_2_ binds to both primary and secondary amines in 2-ampd, forming ammonium carbamate and carbamic acid, respectively. This mechanism was supported by TPD data showing co-desorption of water and CO_2_, as well as DRIFTS measurements revealing the loss of N-H stretching from secondary amines and the emergence of O-H stretching in humid samples.

**FIGURE 7:**
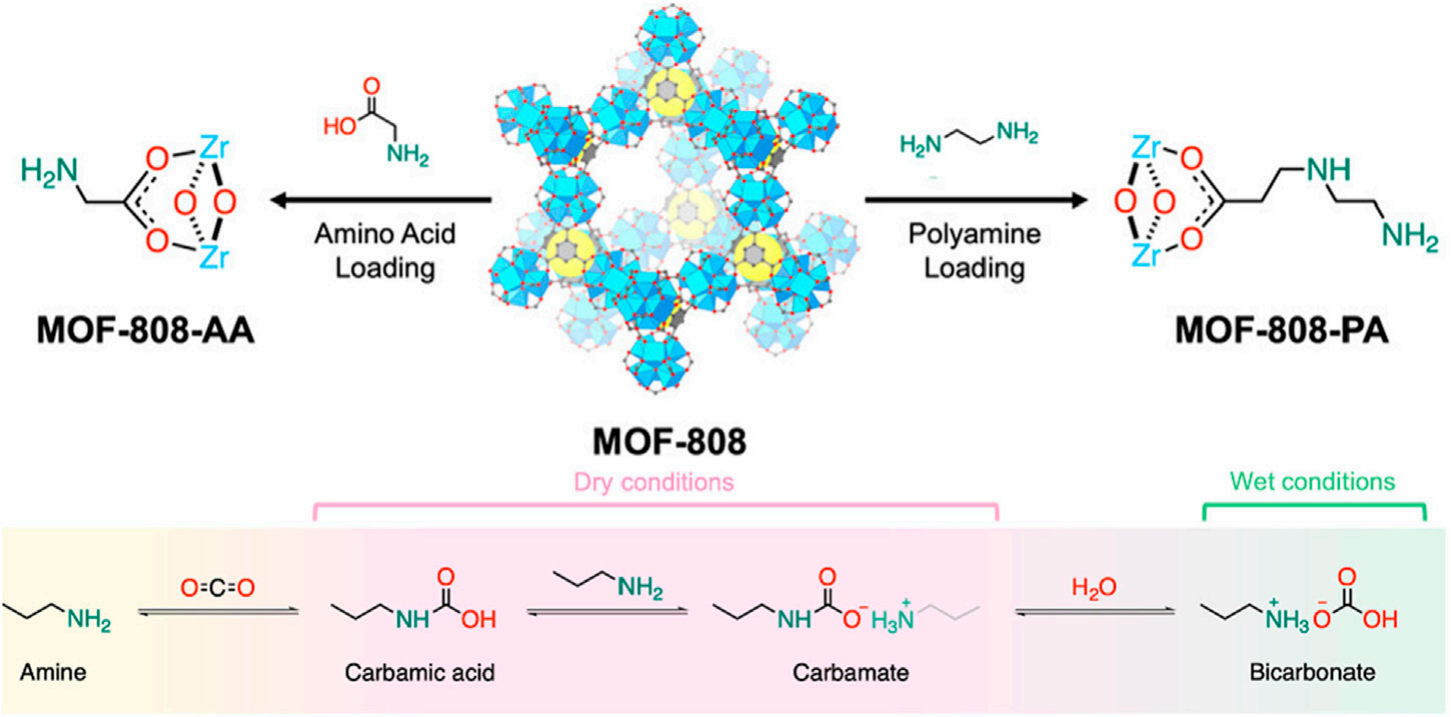
(Top) Two series of amine-functionalized MOF-808: MOF-808-AAs and MOF-808-PAs. (Bottom) Proposed chemisorption mechanism of amines with CO_2_ under dry and wet conditions. Reprinted with permission from Chen et al. (2024). Copyright 2024, American chemical society.

Didas et al. (2014) reported that co-adsorption of CO_2_ and water on mesoporous silica with low amine surface coverage leads to bicarbonate formation. Recently, Lyu et al. (2022) and Chen et al. (2024) both demonstrated that bicarbonate formation within MOFs can enhance CO_2_ uptake. In their studies, they developed amine-functionalized MOF-808 materials: one featuring amino acids coordinated to Zr ions (MOF-808-AAs), first reported by Lyu et al. (2022), and the other incorporating polyamines covalently attached to a chloro-functionalized framework (MOF-808-PAs), reported by Chen et al. (2024), see Figure 7 (top). Both series exhibited improved CO_2_ capture performance under humid conditions for direct air capture of CO_2_, where the CO_2_ concentration is approximately 420 ppm in the atmosphere. At 50% RH, the l-lysine- and tris(3-aminopropyl)amine-functionalized variants exhibited remarkable uptakes of 1.205 and 0.872 mmol/g at 400 ppm CO_2_ and 25°C corresponding to 97% and 75% increases compared to the dry uptakes, respectively. The detailed sorption study using solid-state NMR revealed that for the wet conditions, MOF-808-Lys exhibited a single signal at 167 ppm under 50% RH attributed to ammonium bicarbonate formation. The presence of water enhanced the amine utilization efficiency by forming bicarbonate species (see Figure 7 (bottom)), resulting in increased CO_2_ uptake compared to the dry conditions.

## 3 Conclusion

The presence of water vapor, long considered a challenge in CO_2_ capture, is now recognized as a potential partner in enhancing CO_2_ adsorption performance in certain MOFs. A growing body of experimental and computational studies has revealed several mechanisms through which water can improve CO_2_ uptake, including dipole–quadrupole interactions, water-assisted formation of new adsorption sites, confinement effects via water nanopockets, and ammonium carbamate, carbamic acid and bicarbonate formation at reactive amine sites. These mechanisms, observed in a diverse range of MOFs, such as HKUST-1, MIL-100(Fe), PCN-250, and MOF-808, demonstrate that well-designed frameworks can convert water from a disruptive presence into a cooperative one. While many of these enhancements occur under low or moderate humidity, challenges remain in boosting performance under high relative humidity, where water saturation can reduce pore accessibility. Continued exploration of MOF structures, functional groups, and water-CO_2_ interactions will be key to designing next-generation materials for practical CO_2_ capture, especially in humid environments such as flue gas streams and ambient air. The development of MOFs exhibiting high CO_2_ selectivity, capacity, and stability under humid conditions represents a promising path forward in advancing scalable carbon capture technologies.

## 4 Perspectives

The emerging understanding of water-enhanced CO_2_ capture in MOFs presents an exciting opportunity to rethink the role of moisture in gas capture. While water vapor has traditionally been viewed as a challenge, competing with CO_2_ for adsorption sites and destabilizing frameworks, recent findings demonstrate that, under specific structural and chemical conditions, water can become a cooperative agent that enhances CO_2_ uptake. Mechanisms such as dipole–quadrupole interactions, molecular confinement, water-induced site activation, and bicarbonate formation at amine-functionalized sites have all been shown to improve performance in humid environments. The simple dipole–quadrupole interaction model between water and CO_2_ molecules suggests that positively charged adsorption sites formed via water coordination can promote CO_2_ uptake. If a framework offers an environment that facilitates the creation of such sites, enhanced CO_2_ adsorption can be achieved. Rational incorporation of hydrophilic functional groups, such as μ_2_/μ_3_-OH bridges and open metal sites, can facilitate structured water adsorption, promoting CO_2_ capture. Additionally, frameworks with hierarchical pore structures may provide the spatial freedom to accommodate water and CO_2_ without compromising access to active sites. The “plier effect” and the formation of new reactive sites from water dissociation further suggest that cooperative interactions can be engineered to improve performance under humid conditions.

Despite these advances, significant knowledge gaps remain. For instance, systematic studies of water-enhanced CO_2_ capture across a range of relative humidities are lacking. Likewise, the effect of varying CO_2_ concentrations, such as those relevant to direct air capture, has not been thoroughly explored in humid conditions. Advanced *in situ* techniques, such as solid-state NMR, IR spectroscopy, and X-ray scattering, coupled with multiscale computational modeling, will be vital for unraveling these complex interactions at the molecular level. Furthermore, future research should include a focus on bridging the gap between fundamental discovery and practical deployment. This focus would include improving the stability and regenerability of MOFs under cyclic operation, scaling up synthesis methods, and integrating these materials into realistic gas separation processes, such as post-combustion carbon capture and direct air capture.
